# Effects of sacubitril/valsartan on ventricular remodeling in patients with hypertension and maintenance hemodialysis: a retrospective cohort study

**DOI:** 10.3389/fcvm.2026.1776823

**Published:** 2026-03-03

**Authors:** Lili Jiang, Xu Min, Jing Ran, Yu Zhu, Luping Pan, Bayi Yang, Xue Ran, Ying Ran, Hejun Ding, Jurong Yang, Shaofa Wu

**Affiliations:** 1 Department of Nephrology, The Third Affiliated Hospital of Chongqing Medical University, Chongqing, China; 2 Department of Nephrology, Youyang Hospital, a Branch of The First Affiliated Hospital of Chongqing Medical University, Chongqing, China; 3 Department of Ultrasonography, Youyang Hospital, a Branch of The First Affiliated Hospital of Chongqing Medical University, Chongqing, China

**Keywords:** hypertension, left ventricular hypertrophy, maintenance hemodialysis, sacubitril/valsartan, ventricular remodeling

## Abstract

**Background:**

Left ventricular hypertrophy (LVH), a hallmark of pathological ventricular remodeling, is highly prevalent and strongly predicts mortality in patients undergoing maintenance hemodialysis (MHD). This study aimed to compare the efficacy of sacubitril/valsartan (SV) vs. angiotensin-converting enzyme inhibitors/angiotensin receptor blockers (ACEi/ARB) on ventricular remodeling in hypertensive MHD patients.

**Methods:**

In this single-center retrospective cohort study conducted between January 2023 and June 2025, 111 hypertensive patients undergoing MHD for at least 3 months were divided into SV (*n* = 46) and ACEi/ARB (*n* = 65) groups based on their antihypertensive regimen. The primary endpoint was the change in left ventricular mass index (LVMI) after 6 months. Secondary endpoints included changes in blood pressure, other echocardiographic parameters, NT-proBNP, and safety outcomes.

**Results:**

The mean age was 56.17 ± 13.47 years and 68.5% were male. After 6 months, LVMI significantly decreased in the SV group (−5.52 g/m^2^, 95% CI −9.35 to −1.69, *P* = 0.006) but not in the ACEi/ARB group (1.11 g/m^2^, 95% CI −3.27 to 5.50, *P* = 0.615). Two-way repeated measures ANOVA revealed a significant group × time interaction for LVMI (*P* = 0.033). Both groups achieved significant blood pressure reductions: systolic blood pressure decreased by 9.81 mmHg (*P* < 0.001) in the ACEi/ARB group and by 10.46 mmHg (*P* < 0.001) in the SV group. New-onset intradialytic hypotension occurred in 7 (6.3%) patients and hyperkalemia in 9 (8.1%) patients, with similar incidences between groups and no treatment discontinuations.

**Conclusions:**

Compared to ACEi/ARB, SV is more effective in reversing ventricular remodeling in hypertensive MHD patients.

## Introduction

End-stage renal disease (ESRD) is a global health burden, with maintenance hemodialysis (MHD) serving as the primary modality of renal replacement therapy (1). Despite advances in dialysis technology, the prognosis for MHD patients remains poor, with cardiovascular disease (CVD) accounting for approximately 50% of all deaths (2, 3). Among the spectrum of cardiovascular pathologies, ventricular remodeling is highly prevalent in dialysis patients (4). It serves as a core pathophysiological link and an independent predictor of heart failure, malignant arrhythmias, and sudden cardiac death (5–8).

The prevalence of hypertension in patients with MHD exceeds 80% and is a major driver of ventricular remodeling (9). The pathophysiological mechanisms are complex and distinct from the general population, involving volume overload, chronic activation of the renin-angiotensin-aldosterone system (RAAS), sympathetic nervous system hyperactivity, endothelial dysfunction, and the accumulation of uremic toxins (10, 11). Collectively, these factors contribute to cardiomyocyte hypertrophy and interstitial fibrosis. Although current guidelines emphasize stringent blood pressure (BP) control through the use of RAAS inhibitors, such as angiotensin-converting enzyme inhibitors (ACEi) and angiotensin receptor blockers (ARB), significant clinical challenges remain (12). First, the efficacy of these treatments is often compromised, as ‘uremic cardiomyopathy’ may resist standard RAAS blockade through non-hemodynamic fibrotic pathways (13, 14). Second, safety concerns, particularly regarding the risks of hyperkalemia and the potential loss of residual renal function, frequently lead to cautious or suboptimal dosing (15). Furthermore, hemodynamic fluctuations during dialysis can exacerbate ventricular remodeling (16). Therefore, there is an urgent need for therapeutic strategies that effectively target these pathological pathways while ensuring a superior safety profile.

Sacubitril/valsartan (SV) is a first-in-class angiotensin receptor neprilysin inhibitor (ARNI) composed of the neprilysin inhibitor sacubitril and the angiotensin II receptor blocker valsartan in a 1:1 molar ratio. It exerts its effects through a dual mechanism: the valsartan component blocks ARB receptors, inhibiting the harmful effects of the RAAS; meanwhile, the sacubitril component inhibits neprilysin, increasing the levels of natriuretic peptides and other vasoactive peptides. This enhances their beneficial effects of promoting urinary sodium excretion, vasodilation, anti-fibrosis, and anti-cardiomyocyte hypertrophy (17). Landmark trials in heart failure with reduced ejection fraction (HFrEF) have demonstrated superior reverse remodeling with SV compared with ACEi/ARB (18). Although initially indicated for HFrEF, SV is increasingly utilized for the management of essential hypertension due to its potent antihypertensive effects and potential for organ protection, even in patients without overt heart failure (19). Emerging evidence also suggests mortality and hospitalization benefits in patients requiring hemodialysis (14, 20). However, real-world data comparing SV with ACEi/ARB specifically for ventricular remodeling in hypertensive MHD patients without overt heart failure remain scarce.

This study aimed to evaluate the efficacy of SV compared with ACEi/ARB therapy on ventricular remodeling and blood pressure control in patients with MHD and hypertension in a real-world setting.

## Methods

### Study design and participants

This single-center, retrospective cohort study was conducted at the Hemodialysis Center of Youyang Hospital, The First Affiliated Hospital of Chongqing Medical University, from 1 January 2023 to 30 June 2025. Patients were stratified into the SV group and the ACEi/ARB group according to their prescribed medication regimen initiated at baseline. The decision to prescribe SV or ACEi/ARB was made by the treating physician based on blood pressure control needs and clinical judgment regarding organ protection. The study protocol adhered to the Declaration of Helsinki and was approved by the institutional ethics committee. The requirement for informed consent was waived due to the retrospective design.

Inclusion Criteria: (1) Age ≥18 years; (2) Regular hemodialysis for ESRD for ≥ 3 months (4 h/session, 2–3 times/week); (3) Diagnosis of hypertension with new initiation or switch to either SV or ACEi/ARB drugs for ≥6 months; and (4) Availability of complete echocardiographic and clinical laboratory data at baseline and 6-month follow-up.

Exclusion Criteria: (1) NYHA class IV heart failure or hospitalization for acute heart failure within the preceding 6 months; (2) significant structural heart disease (severe valvular disease, hypertrophic cardiomyopathy, or pericardial disease); (3) severe comorbidities, including active malignancy, severe infection, or hepatic dysfunction (Child-Pugh class C); (4) history of angioedema; and (5) incomplete clinical data.

The diagnosis of hypertensive nephropathy was established clinically based on a composite of criteria: (1) a documented history of long-standing hypertension preceding the onset of kidney dysfunction; (2) evidence of hypertension-mediated target-organ damage, such as hypertensive retinopathy and/or left ventricular hypertrophy (LVH); (3) the absence of features suggestive of an alternative primary renal disease, including active urinary sediment or nephrotic-range proteinuria, when such data were available; and (4) the exclusion of other plausible etiologies based on a comprehensive chart review (21). In this retrospective cohort, kidney biopsy was not routinely performed and was generally reserved for atypical clinical presentations.

Patients were stratified into the SV group and the ACEi/ARB group according to their prescribed medication regimen. The initial oral dose of SV was 50 mg twice daily, with titration every 2–4 weeks up to a maximum of 200 mg twice daily, according to blood pressure response and tolerability. The final dosage of SV in the study group is detailed in Supplementary Table S1. The numbers of MHD patients with hypertension who received 50 mg twice daily, 100 mg twice daily, and 200 mg twice daily were 6 (13.04%), 28 (60.87%), and 12 (26.09%), respectively. The oral dose of ACEi/ARB drugs for patients was the tolerable dose. Concomitant antihypertensive medications were generally maintained at stable doses throughout the observation period to minimize confounding variables, unless dose adjustments were clinically required due to safety concerns.

### Data collection and outcomes

Clinical and demographic data were retrieved from the Hospital Information System (HIS) and Laboratory Information System (LIS). Baseline characteristics included age, gender, dialysis vintage, primary etiology of ESRD, comorbidities (e.g., diabetes mellitus, coronary heart disease), and concomitant medications.

### Laboratory assessments

Laboratory indicators were collected at baseline and at the 6-month follow-up. These included N-terminal pro-brain natriuretic peptide (NT-proBNP), serum creatinine, blood urea nitrogen, serum potassium, serum calcium, serum phosphate, intact parathyroid hormone, hemoglobin, albumin, high-sensitivity C-reactive protein (hs-CRP), alanine aminotransferase (ALT), aspartate aminotransferase (AST), β2-microglobulin, spKt/v, and serum ferritin.

### Echocardiographic and blood pressure measurements

Transthoracic echocardiography was performed by experienced sonographers using a standardized protocol at baseline and at 6 months (±2 weeks) after initiation of treatment. All measurements were taken in accordance with the current recommendations of the American Society of Echocardiography (ASE) and the European Association of Cardiovascular Imaging (22). Key parameters included left ventricular end-diastolic diameter (LVEDd), left ventricular end-systolic diameter (LVESd), interventricular septum thickness in diastole (IVSTd), and posterior wall thickness at end-diastole (PWTd).

Left ventricular mass (LVM) was calculated using the Devereux-modified ASE cube formula:

$$\mathrm{LVM}(g)\;=\;0.8\;\times \;\{1.04\;[{\left(\mathrm{LVEDd}+\mathrm{IVSTd}+\mathrm{PWTd}\right)}^{3}-\;{\left(\mathrm{LVEDd}\right)}^{3}]\}\;+\;0.6$$

LVM was then indexed to body surface area (Du Bois formula) to obtain the left ventricular mass index (LVMI, g/m^2^).

Left ventricular ejection fraction (LVEF) was measured using the biplane Simpson's method. All other linear dimensions were measured in the parasternal long-axis view at end-diastole.

Pre-dialysis blood pressure was measured in the sitting position using a calibrated electronic sphygmomanometer after at least 5 min of rest. At both baseline and 6-month follow-up, blood pressure was recorded immediately before three consecutive hemodialysis sessions, and the average of these three measurements was used for analysis.

### Primary endpoint

The primary efficacy endpoint was the change in LVMI from baseline to 6 months.

### Secondary endpoints

The secondary endpoints including the LVEDd (mm), LVESd (mm), IVSTd (mm), PWTd (mm), and LVEF (%). Other secondary objectives included the changes in NT-proBNP, systolic blood pressure (SBP), diastolic blood pressure (DBP) and pulse pressure.

### Safety endpoints

Safety and tolerability were assessed by monitoring adverse events (AEs), specifically symptomatic hypotension, intradialytic hypotension, angioedema, cough, hepatic dysfunction, and hyperkalemia (serum potassium >5.5 mmol/L). Intradialytic hypotension was defined as a decrease in SBP ≥20 mmHg or mean arterial pressure ≥10 mmHg during dialysis that was accompanied by symptoms (e.g., cramping, headache, lightheadedness, vomiting, chest pain) or required intervention (e.g., reduced ultrafiltration, fluid administration, or blood flow reduction).

### Statistical analysis

All statistical analyses and diagrams were conducted using GraphPad Prism, version 9.0, and SPSS version 27.0 software (IBM Corp., Armonk, NY). Categorical variables are presented as percentages (%) and counts, while normally—distributed continuous variables are reported as the means ± standard deviations (SD). Non—normally distributed continuous variables are represented by the median and interquartile range (IQR). To compare the mean values before and after treatment, a paired *t*-test was used if the differences followed a normal distribution. Conversely, the Wilcoxon signed—rank test for paired samples was utilized when the differences did not adhere to a normal distribution. To compare longitudinal changes between groups, we applied a two-way repeated-measures ANOVA and reported the treatment group × time interaction *P* value. Multiple linear regression analysis was performed to identify independent predictors of changes in LVMI. Variables were selected for the multivariate model based on clinical relevance and significant baseline differences observed between the groups. No adjustment for multiplicity was applied for secondary endpoints. To further address potential selection bias and baseline imbalances, a sensitivity analysis was performed using propensity score (PS) adjustment. A logistic regression model including age, sex, dialysis vintage, hypertensive nephropathy, baseline LVMI, SBP, and baseline use of CCBs and α-blockers was used to calculate the PS for receiving SV. This PS was then included as a covariate in the linear regression model for the change in LVMI. A two-tailed *P*-value < 0.05 was considered statistically significant.

## Results

### Baseline characteristics

**Figure 1:**
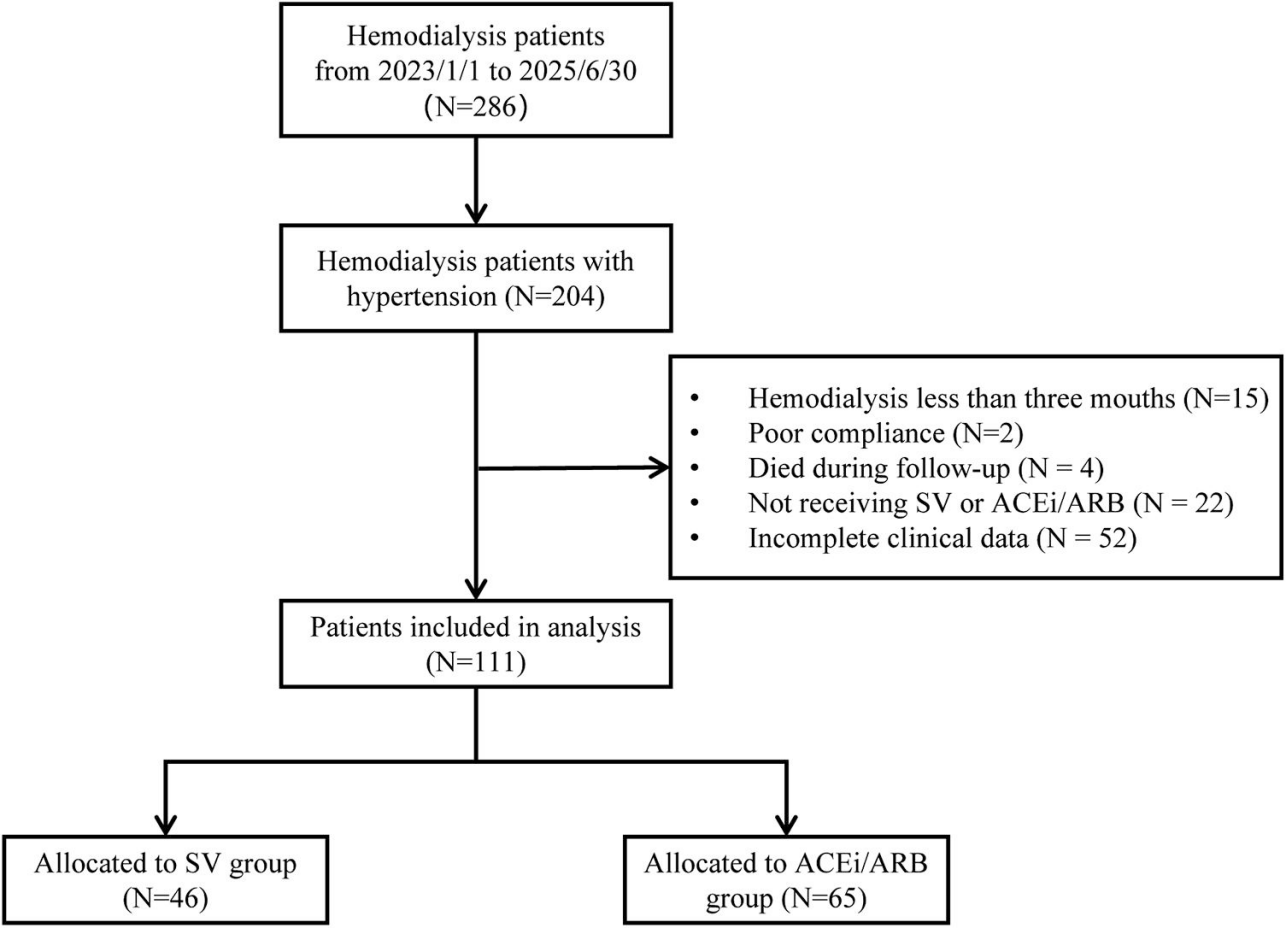
Flow diagram for patient inclusion and exclusion in the study. SV, sacubitril/valsartan; ACEi/ARB, angiotensin-converting enzyme inhibitors/angiotensin receptor blockers.

The study screened 286 MHD patients from our hospital between January 1, 2023, and June 30, 2025. Among them, 204 were diagnosed with hypertension. According to the exclusion criteria, we excluded 15 patients with a dialysis duration of less than three months, 4 patient who died during follow-up, 22 patients not taking SV or ACEi/ARB class antihypertensive drugs, and 52 patients with incomplete clinical data were excluded. Finally, 111 patients were included. The cohort included 46 patients in the SV group and 65 in the ACEi/ARB group (Figure 1).

Baseline characteristics are summarized in Table 1. The mean age was 56.17 ± 13.47 years, and 68.5% were male. The average BMI was 22.20 ± 3.37 kg/m^2^, and the median dialysis duration was 45.50 (21.0–78.25) months. The main cause of ESRD was glomerulonephritis (35.1%), followed by diabetic nephropathy (25.2%), hypertensive nephropathy (21.6%), and other causes (18.0%). Most patients (91.0%) underwent dialysis three times per week. Regarding other medications, 58.6% of patients received calcium channel blockers, 28.8% received β-blockers, and 24.3% received *α*-blockers. Notably, dialysis vintage was longer in the SV group compared to the ACEi/ARB group (64.50 vs. 34.00 months; *P* = 0.010). The proportion of patients with hypertensive nephropathy was also higher in the SV group (34.8% vs. 12.3%; *P* = 0.005). Additionally, the use of calcium channel blockers (80.4% vs. 43.1%) and alpha-blockers (43.5% vs. 10.8%) was significantly more frequent in the SV group. Baseline echocardiographic parameters were comparable between the two groups (Supplementary Table S2).

**Table 1:** Patients demographic and baseline clinical characteristics.

Variables	All patients (*n* = 111)	ACEi/ARB treated patients (*n* = 65)	Sacubitril/Valsartan treated patients (*n* = 46)	*P*
Age, years	56.17 ± 13.47	58.39 ± 13.02	55.09 ± 11.14	0.201
Male gender, *n* (%)	76 (68.5)	44 (67.7)	32 (69.6)	0.834
BMI, (Kg/m^2^)	22.20 ± 3.37	22.12 ± 3.40	22.30 ± 3.36	0.796
Dialysis age, (months)	45.50 (21.0–78.25)	34.00 (19.25–53.50)	64.50 (35.75–84.00)	0.010
Causes of ESRD				
Glomerulonephritis, *n* (%)	39 (35.1)	26 (40.0)	13 (28.3)	0.202
Diabetes related kidney disease, *n* (%)	28 (25.2)	17 (26.2)	11 (23.9)	0.789
Hypertensive nephropathy, *n* (%)	24 (21.6)	8 (12.3)	16 (34.8)	0.005
Others, *n* (%)	20 (18.0)	14 (21.5)	6 (13.0)	0.251
Frequency of dialysis				
3 times per week, *n* (%)	101 (91.0)	59 (90.8)	42 (91.3)	0.923
2 times per week, *n* (%)	10 (9.0)	6 (9.2)	4 (8.7)	0.923
Other medications				
calcium channel blocker, *n* (%)	65 (58.6)	28 (43.1)	37 (80.4)	<0.001
β-blocker, *n* (%)	32 (28.8)	16 (24.6)	16 (34.8)	0.244
α-blocker, *n* (%)	27 (24.3)	7 (10.8)	20 (43.5)	<0.001
Laboratory indices				
blood urea nitrogen, (mmol/L)	19.92 (16.71–23.13)	19.92 (15.36–23.06)	19.97 (17.21–23.78)	0.300
serum creatinine, (μmol/L)	984.15 (745.95–1,240.10)	953.85 (715.93–1,193.38)	1,007.50 (847.80–1,300.25)	0.056
serum uric acid, (μmol/L)	468.00 (383.00–521.00)	464.50 (361.00–527.75)	469.50 (390.75–519.00)	0.547
spKt/v	1.29 (1.16–1.45)	1.29 (1.13–1.51)	1.29 (1.16–1.42)	0.808
β2-microglobulin, (mg/L)	35.29 ± 9.14	33.64 ± 9.24	37.58 ± 8.58	0.012
Serum calcium, (mmol/L)	2.18 ± 0.23	2.15 ± 0.24	2.23 ± 0.21	0.057
Serum phosphate, (mmol/L)	1.64 (1.35–2.00)	1.60 (1.28–2.07)	1.67 (1.44–1.92)	0.557
Ferritin, (ng/mL)	55.65 (29.55–137.18)	80.80 (35.88–159.00)	40.95 (25.35–82.08)	0.006
Serum potassium, (mmol/L)	4.61 ± 0.70	4.52 ± 0.72	4.74 ± 0.64	0.104
Hemoglobin, (g/L)	113.82 ± 15.95	113.11 ± 17.19	114.80 ± 14.18	0.530
Parathyroid hormone, (pg/ml)	273.50 (175.55–424.25)	292.55 (157.25–395.48)	260.50 (187.25–477.45)	0.501
Albumin, (g/L)	39.50 (37.38- 41.00)	39.60 (37.43–40.95)	39.45 (37.23–41.35)	0.883

Continuous values are documented by Mean ± SD or Median (interquartile); Categorical variables are recorded by *n* (percentage). ACEi/ARB, angiotensin-converting enzyme inhibitors/angiotensin receptor blockers; BMI, body mass index; ESRD, end-stage renal disease; spKt/v, single-pool kt/V.

### Primary endpoint

**Figure 2:**
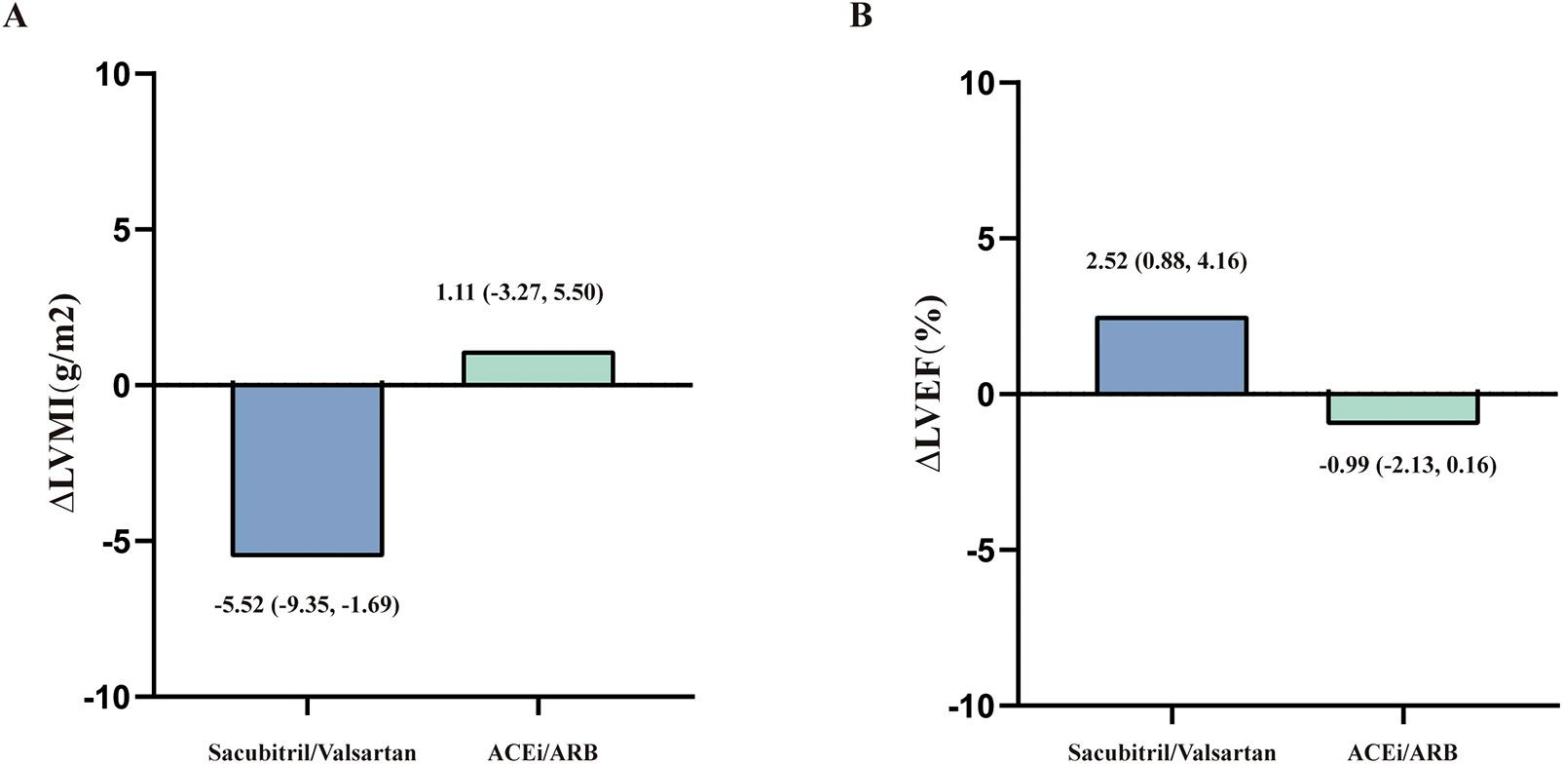
Comparison of the treatment effect between groups, expressed as the mean change from baseline to 6 months for left ventricular mass Index (ΔLVMI, **A**) and left ventricular ejection fraction ΔLVEF, **B**).

In the SV group, LVMI significantly decreased from 139.08 ± 36.38 g/m^2^ at baseline to 133.56 ± 31.68 g/m^2^ at 6 months (*P* = 0.006, Table 2). In contrast, the ACEi/ARB group showed no significant improvement in LVMI, which changed from 139.82 ± 39.96 to 140.93 ± 37.51 g/m^2^ (*P* = 0.615, Table 2). Two-way repeated-measures ANOVA (Table 3) showed that although the main effect of time (*P* = 0.153) and the main effect of group (*P* = 0.557) on LVMI were not statistically significant, the Time × Group interaction was significant (*P* = 0.033). This interaction suggests that the reduction in LVMI over time was significantly different between the two treatment arms, with a notable advantage observed in the SV group. Figure 2A displays the change in LVMI between the two groups [−5.52 (−9.35 to −1.69) vs. 1.11 (−3.27 to 5.50)].

**Table 2:** Changes in left ventricular mass and function in maintenance hemodialysis patients with hypertension.

Parameter and group	Baseline	Follow-up	ΔMean (95%CI)	*P*
LVMI, g/m^2^				
ACEi/ARB	139.82 ± 39.96	140.93 ± 37.51	1.11 (−3.27 to 5.50)	0.615
Sacubitril/Valsartan	139.08 ± 36.38	133.56 ± 31.68	−5.52 (−9.35 to −1.69)	0.006
LVEDd, mm				
ACEi/ARB	49.29 ± 6.13	49.54 ± 5.79	0.25 (−0.77 to 1.26)	0.629
Sacubitril/Valsartan	50.96 ± 5.69	50.39 ± 4.70	−0.57 (−1.32 to 0.19)	0.138
LVESd, mm				
ACEi/ARB	33.06 ± 6.67	32.08 ± 6.42	−0.99 (−2.01 to 0.09)	0.072
Sacubitril/Valsartan	34.63 ± 7.19	34.24 ± 6.10	−0.39 (−2.15 to 1.37)	0.656
PWTd, mm				
ACEi/ARB	10.85 ± 1.65	10.97 ± 1.55	0.12 (−0.16 to 0.41)	0.387
Sacubitril/Valsartan	11.19 ± 1.75	11.11 ± 1.35	−0.08 (−0.41 to 0.24)	0.600
IVSTd, mm				
ACEi/ARB	11.69 ± 1.85	11.60 ± 1.58	−0.09 (−0.39 to 0.20)	0.531
Sacubitril/Valsartan	11.78 ± 1.64	11.52 ± 1.52	−0.27 (−0.63 to 0.10)	0.147
LVEF, %				
ACEi/ARB	60.43 ± 9.16	59.45 ± 10.09	−0.99 (−2.13 to 0.16)	0.091
Sacubitril/Valsartan	60.28 ± 7.26	62.80 ± 8.88	2.52 (0.88 to 4.16)	0.003
NT-pro BNP, Log				
ACEi/ARB	3.62 ± 0.65	3.45 ± 0.59	−0.16 (−0.28 to −0.04)	0.008
Sacubitril/Valsartan	3.55 ± 0.55	3.39 ± 0.45	−0.16 (−0.30 to −0.02)	0.030

CI, confidence interval; LVMI, left ventricular mass index; ACEi/ARB, angiotensin-converting enzyme inhibitors/angiotensin receptor blockers; LVEDd, left ventricular end-diastolic dimension; LVESd, left ventricular end-systolic diameter; PWTd, posterior wall thickness at end-diastole; IVSTd, interventricular septum thickness in diastole; LVEF, Left ventricular ejection fraction.

**Table 3:** Two-way ANOVA results for left ventricular mass and function in maintenance hemodialysis patients with hypertension.

Parameter and group	Effect	F-value	*P*
LVMI, g/m^2^			
	Group	0.348	0.557
	Time	2.07	0.153
	Group × Time interaction	4.69	0.033
LVEF, %			
	Group	0.920	0.340
	Time	2.53	0.114
	Group × Time interaction	13.17	<0.001
LVEDd, mm			
	Group	1.471	0.228
	Time	0.220	0.640
	Group × Time interaction	1.422	0.236
LVESd, mm			
	Group	2.523	0.115
	Time	1.994	0.161
	Group × Time interaction	0.371	0.544
PWTd, mm			
	Group	7.003	0.009
	Time	0.032	0.859
	Group × Time interaction	0.931	0.337
IVSTd, mm			
	Group	0.001	0.978
	Time	2.402	0.124
	Group × Time interaction	0.562	0.455
NT-pro BNP, Log			
	Group	0.460	0.499
	Time	12.075	<0.001
	Group × Time interaction	0.002	0.967

LVMI, left ventricular mass index; LVEF, Left ventricular ejection fraction; LVEDd, left ventricular end-diastolic dimension; LVESd, left ventricular end-systolic diameter; PWTd, posterior wall thickness at end-diastole; IVSTd, interventricular septum thickness in diastole.

### Secondary endpoints

LVEF changed from 60.43 ± 9.16% to 59.45 ± 10.09% in the ACEi/ARB group (*P* = 0.091), and from 60.28 ± 7.26% to 62.80 ± 8.88% in the SV group (*P* = 0.003; Table 2). ANOVA demonstrated a significant Group × Time interaction for LVEF (*P* < 0.001; Table 3). Figure 2B displays the change in LVEF between the two groups [2.52 (0.88–4.16) vs. −0.99 (−2.13 to 0.16)]. Other echocardiographic indices, including LVEDd, LVESd, IVSTd, and PWTd, did not change significantly over 6 months in either group (*P* > 0.05; Table 2). Consistently, ANOVA showed no significant group  ×  time interaction for LVEDd, LVESd, PWTd, IVSTd or Log-transformed NT-proBNP (*P* > 0.05; Table 3).

The ACEi/ARB group achieved a significant reduction in SBP of 9.81 mmHg (*P* < 0.001), DBP of 3.19 mmHg (*P* = 0.035) and pulse pressure of 6.63 mmHg (*P* = 0.002, Table 4). Similarly, the SV group showed significant decreases in SBP (−10.46 mmHg, *P* < 0.001), DBP (−4.13 mmHg, *P* = 0.021) and pulse pressure (−6.32 mmHg, *P* = 0.004, Table 4).

**Table 4:** Comparison of blood pressure changes in maintenance hemodialysis patients with hypertension.

Parameter and group	Baseline	Follow-up	ΔMean (95%CI)	*P*
SBP, mmHg				
ACEi/ARB	150.49 ± 12.51	140.68 ± 13.58	−9.81 (−14.21 to −5.42)	<0.001
Sacubitril/Valsartan	147.72 ± 14.62	137.26 ± 11.67	−10.46 (−15.50 to−5.42)	<0.001
DBP, mmHg				
ACEi/ARB	86.26 ± 9.70	83.08 ± 9.80	−3.19 (−6.13 to −0.24)	0.035
Sacubitril/Valsartan	87.24 ± 10.85	83.11 ± 10.75	−4.13 (−7.62 to −0.64)	0.021
Pulse pressure, mmHg				
ACEi/ARB	64.23 ± 13.65	57.60 ± 15.33	−6.63 (−10.82 to −3.17)	0.002
Sacubitril/Valsartan	60.48 ± 13.42	54.15 ± 11.37	−6.32 (−10.49 to −2.16)	0.004

CI, confidence interval; SBP, systolic blood pressure; ACEi/ARB, angiotensin-converting enzyme inhibitors/angiotensin receptor blockers; DBP, diastolic blood pressure.

Log-transformed NT-proBNP levels also declined significantly in both cohorts. The SV group showed a reduction from 3.55 ± 0.55 to 3.39 ± 0.45 (*P* = 0.030), and the ACEi/ARB group showed a reduction from 3.62 ± 0.65 to 3.45 ± 0.59 (*P* = 0.008, Table 2).

### Multiple linear regression and sensitivity analysis

After adjusting for potential confounders including age, male gender, dialysis vintage, hypertensive nephropathy, concomitant CCBs, concomitant β-blockers, baseline LVMI, and baseline SBP, treatment with SV remained a significant independent predictor of LVMI improvement (*P* = 0.023; Table 5). Baseline LVMI was also significantly associated with changes in LVMI (*P* < 0.001; Table 5).

**Table 5:** Results of multiple regression analysis for factors affecting left ventricular mass index changes.

Parameter	Model 1: β (95% CI)	*P*	Model 2: β (95% CI)	*P*
Sacubitril/Valsartan	−7.34 (−13.64 to −1.03)	0.023	−8.20 (−14.69 to −1.71)	0.014
Age	0.14 (−0.09 to 0.37)	0.229	0.17 (−0.07 to 0.40)	0.163
Male gender	−1.25 (−7.52 to 5.03)	0.694	−1.24 (−7.50 to 5.03)	0.696
Dialysis age	−0.01 (−0.09 to 0.07)	0.730	−0.04 (−0.13 to 0.05)	0.412
Hypertensive nephropathy	5.34 (−2.03 to 12.71)	0.154	4.87 (−2.55 to 12.28)	0.196
calcium channel blocker	−1.23 (−8.00 to 5.54)	0.719	−5.87 (−16.68 to 4.94)	0.284
β-blocker	6.09 (−0.50 to 12.68)	0.070	5.20 (−1.59 to 11.98)	0.132
Baseline LVMI	−0.17 (−0.24 to −0.09)	<0.001	−0.17 (−0.24 to −0.09)	<0.001
Baseline SBP	0.06 (−0.16 to 0.28)	0.597	0.14 (−0.12 to 0.40)	0.298
Propensity score	—	—	14.21 (−11.62 to 40.03)	0.278

Model 1 was adjusted for age, sex, dialysis vintage, hypertensive nephropathy, baseline use of calcium channel blockers and β-blockers, baseline LVMI, and baseline SBP. Model 2 additionally included the propensity score (PS). CI, confidence interval; LVMI, left ventricular mass index; SBP, systolic blood pressure.

In the sensitivity analysis using propensity score adjustment, the propensity score was included in the regression model. The results confirmed that SV treatment was significantly associated with a greater reduction in LVMI (*β* = −8.20, 95% CI −14.69 to −1.71, *P* = 0.014) (Table 5).

### Safety outcomes

The AEs are summarized in Table 6. The overall incidence of new-onset intradialytic hypotension was 6.3% (7 patients), with comparable rates between the ACEi/ARB (6.1%, *n* = 4) and SV (6.5%, *n* = 3) groups. Hyperkalemia occurred in 8.1% of patients (*n* = 9), including 5 (7.6%) in the ACEi/ARB group and 4 (8.6%) in the SV group. No cases of angioedema or cough were reported. Importantly, no patients required dose reduction or treatment discontinuation due to adverse events.

**Table 6:** Adverse events of patients in maintenance hemodialysis with hypertension.

Parameter	Overall	ACEi/ARB (*n* = 65)	Sacubitril/ Valsartan (*n* = 46)
New-onset intradialytic hypotension^a^, *n* (%)	7 (6.3)	4 (6.1)	3 (6.5)
Hyperkalemia (>5.5 mmol/L), *n* (%)	9 (8.1)	5 (7.6)	4 (8.6)
Cough, *n* (%)	0 (0)	0 (0)	0 (0)
Angio-edema, *n* (%)	0 (0)	0 (0)	0 (0)

ACEi/ARB, angiotensin-converting enzyme inhibitors/angiotensin receptor blockers.

^a^ Intradialytic hypotension defined as decrease in systolic blood pressure ≥20 mmHg or mean blood pressure ≥10 mmHg during dialysis treatment with associated symptoms (cramping, headache, lightheadedness, vomiting, or chest pain) or need for intervention (reduction in ultrafiltration, administration of fluids or blood pump flow reduction).

## Discussion

This study offers real-world evidence on the differential efficacy of SV vs. ACEi/ARB in MHD patients. While both treatments achieved effective BP control, only SV significantly reversed ventricular remodeling. Importantly, this differential effect was supported by a significant group × time interaction for LVMI in the ANOVA model. Crucially, this benefit occurred even in patients with a longer dialysis vintage, suggesting that SV exerts distinct cardioprotective effects in uremic cardiomyopathy.

Patients with ESRD exhibit a significantly elevated risk of cardiovascular mortality, primarily due to uremic cardiomyopathy. This condition is characterized by LVH, extensive fibrosis, and diastolic dysfunction (23, 24). LVH is the most prevalent cardiovascular abnormality observed in CKD and ESRD, serving as the strongest independent predictor of cardiovascular mortality within this population (25, 26). In patients undergoing MHD, such pathological ventricular remodeling is closely associated with chronic volume and pressure overload, as well as maladaptive neuroendocrine activation (13). In this context, our study demonstrates that SV significantly reduces LVMI, indicating a reversal of adverse remodeling. These results align with previous data from heart failure populations and recent meta-analyses in nephrology (5, 27, 28). Importantly, our findings extend this evidence by providing retrospective real-world data specifically in hypertensive MHD patients. Notably, the association between SV treatment and LVMI reduction remained significant after multivariable adjustment in the linear regression model.

The superiority of SV in reducing LVMI likely reflects its dual mechanism of action. In addition to inhibiting the RAAS, SV also inhibits neprilysin, thereby enhancing the natriuretic peptide system (17). The natriuretic peptide system, particularly through atrial natriuretic peptide (ANP) and B-type natriuretic peptide (BNP), exerts multiple cardioprotective effects beyond their well-known diuretic and vasodilatory properties (29). These peptides directly inhibit cardiomyocyte hypertrophy by antagonizing the pro-hypertrophic signaling pathways activated by angiotensin II and endothelin-1. Additionally, natriuretic peptides suppress myocardial fibrosis by inhibiting cardiac fibroblast proliferation and reducing collagen synthesis. They also exert anti-inflammatory effects by modulating immune cell function and reducing myocardial inflammation. Furthermore, natriuretic peptides improve myocardial energetics by enhancing mitochondrial function and fatty acid oxidation in cardiomyocytes. Through this combined modulation of RAAS and natriuretic peptide pathways, SV can more comprehensively counteract key mechanisms of myocardial remodeling compared to ACEi or ARB therapy alone.

A critical finding of our study is the dissociation between blood pressure reduction and LVMI improvement. Although both regimens significantly reduced BP and the magnitude of SBP reduction was similar between groups, only SV led to significant regression of LVMI. In the dialysis population, blood pressure is strongly modulated by interdialytic volume status, dry-weight setting, and ultrafiltration, so clinic blood pressure is an imperfect surrogate for long-term hemodynamic load or myocardial stress (9, 11, 30). Moreover, accumulating experimental and clinical data indicate that uremic cardiomyopathy is driven by non-hemodynamic mechanisms, such as inflammation, oxidative stress, and profibrotic signaling, which are inadequately addressed by standard RAAS blockade (13, 14, 31). By combining RAAS inhibition with augmentation of the natriuretic peptide system, sacubitril/valsartan more effectively targets these molecular pathways (17, 32). Given the significant prognostic impact of LVH in ESRD, our findings support a therapeutic shift in hypertensive MHD patients from a sole focus on achieving blood pressure targets toward actively reversing target-organ damage.

Safety remains a primary concern for nephrologists when prescribing RAAS inhibitors. In this study, both drug groups demonstrated good safety and tolerability. A total of 16 adverse events were reported. Symptomatic hypotension occurred in 7 cases (mostly during the initial titration) and was alleviated by adjusting dry weight and concomitant medications. Hyperkalemia occurred in 9 cases (4 in the SV group, 5 in the ACEi/ARB group). All cases were transient and rapidly corrected by dietary adjustments or optimizing dialysis, without leading to treatment interruption. The comparable incidence of hyperkalemia between groups is a reassuring finding for clinicians concerned about the safety of ARNI in anuric patients.

## Limitations

This study has several limitations. First, as a single-center retrospective study, the sample size is relatively limited, and selection bias or unmeasured confounding factors may exist. Specifically, the imbalance in dialysis vintage was noted; however, the fact that the group with longer disease duration achieved better remodeling outcomes strengthens the validity of the observed drug effect. Second, while a 6-month follow-up is sufficient to observe structural changes, it is insufficient to evaluate hard endpoints such as cardiovascular mortality. Third, we did not correct for multiplicity in our secondary endpoint analyses, and the multivariable regression model includes several covariates relative to the sample size, which introduces a risk of overfitting. Fourth, blood pressure was assessed using pre-dialysis measurements, which may not accurately reflect the interdialytic hemodynamic burden or the nocturnal blood pressure dipping. Furthermore, as a retrospective real-world study, examinations were performed by different sonographers without a formal assessment of inter-observer variability (ICC). This may introduce measurement variability. Finally, the echocardiographic assessment was conducted using conventional two-dimensional measurements. More advanced imaging modalities, such as speckle-tracking echocardiography and cardiac magnetic resonance imaging, were not routinely employed and could have offered a more comprehensive evaluation of ventricular remodeling.

## Conclusion

In conclusion, SV demonstrated significantly greater efficacy than ACEi/ARB therapy in reversing ventricular remodeling in MHD patients with hypertension. These findings support the utilization of SV as a therapeutic strategy for cardiovascular protection in the MHD population.

## Data Availability

The original contributions presented in the study are included in the article/Supplementary Material, further inquiries can be directed to the corresponding authors.
