# Supplementary material for: Effects of sacubitril/valsartan on ventricular remodeling in patients with hypertension and maintenance hemodialysis: a retrospective cohort study
Source: Front Cardiovasc Med. 2026 Mar 3;13:1776823. doi: 10.3389/fcvm.2026.1776823 (PMC12992314; doi:10.3389/fcvm.2026.1776823)
Supplement: Supplementary file 1 [file Datasheet1.docx]

**Table S1. Final dose of sacubitril/valsartan in maintenance hemodialysis with hypertension.**

| Final dose | Number of patients (%) |
| --- | --- |
| 50 mg twice daily | 6/46 (13.04%) |
| 100 mg twice daily | 28/46 (60.87%) |
| 200 mg twice daily | 12/46 (26.09) |

**Table S2. Comparison of baseline echocardiographic parameters between treatment groups.**

| Parameter | ACEi/ARB (n = 65) | Sacubitril/Valsartan (n = 46) | *P* |
| --- | --- | --- | --- |
| LVMI, g/m2 | 139.82 ± 39.96 | 139.08 ± 36.38 | 0.919 |
| LVEDd, mm | 49.29 ± 6.13 | 50.96 ± 5.69 | 0.150 |
| LVESd, mm | 33.06 ± 6.67 | 34.63 ± 7.19 | 0.240 |
| PWTd, mm | 10.85 ± 1.65 | 11.19 ± 1.75 | 0.048 |
| IVSTd, mm | 11.69 ± 1.85 | 11.78 ± 1.64 | 0.781 |
| LVEF, % | 60.43 ± 9.16 | 60.28 ± 7.26 | 0.927 |

Abbreviations:ACEi/ARB, angiotensin-converting enzyme inhibitors/angiotensin receptor blockers; LVMI, left ventricular mass index; LVEDd, left ventricular end-diastolic dimension; LVESd, left ventricular end-systolic diameter; PWTd, posterior wall thickness at end-diastole; IVSTd, interventricular septum thickness in diastole; LVEF, Left ventricular ejection fraction.
